# A_2B_AR-Mediated Antiproliferative and Anticancer Effects of Okhotoside A_1_-1 in Monolayer and 3D Culture of Human Breast Cancer MDA-MB-231 Cells

**DOI:** 10.3390/md23120456

**Published:** 2025-11-27

**Authors:** Ekaterina A. Chingizova, Ekaterina S. Menchinskaya, Ekaterina A. Yurchenko, Elena A. Zelepuga, Evgeny A. Pislyagin, Liliana E. Nesterenko, Sergey A. Avilov, Vladimir I. Kalinin, Dmitry L. Aminin, Alexandra S. Silchenko

**Affiliations:** 1 G.B. Elyakov Pacific Institute of Bioorganic Chemistry, Far Eastern Branch of the Russian Academy of Sciences, Pr. 100-Letya Vladivostoka 159, 690022 Vladivostok, Russia; ekaterinamenchinskaya@gmail.com (E.S.M.); eyurch@piboc.dvo.ru (E.A.Y.); zel@piboc.dvo.ru (E.A.Z.); pislyagin@hotmail.com (E.A.P.); nesterenko_le@piboc.dvo.ru (L.E.N.); kalininv@piboc.dvo.ru (V.I.K.); daminin@piboc.dvo.ru (D.L.A.); 2 Department of Biomedical Science and Environmental Biology, Kaohsiung Medical University, Kaohsiung 80708, Taiwan

**Keywords:** triterpene glycosides, triple-negative breast cancer (TNBC), MDA-MB-231, anticancer activity, cell cycle, apoptosis, molecular docking, adenosine receptors, A_2B_AR, antagonist, MAPK

## Abstract

The aim of this study is to investigate the A_2B_AR-dependence of okhotoside A_1_-1 cytotoxic and antiproliferative action on triple-negative MDA-MB-231 breast cancer cells using monolayer and 3D culture approaches. Earlier triterpene glycoside okhotoside A_1_-1 (Okh) was isolated from the sea cucumbers *Cucumaria djakonovi* and *C. conicospermium* and its selective cytotoxicity against MDA-MB-231 vs. non-tumorigenic MCF-10A cells was reported. Now it has been found that the A_2B_ adenosine receptor (A_2B_AR) is one of the molecular targets for Okh and its antiproliferative effect is A_2B_AR-dependent. Molecular docking studies suggested a unique behavior for Okh demonstrating two highly probable binding modes with comparable affinity, when the aglycone is immersed in the binding pocket, or alternatively, the carbohydrate moiety occupies the site. The glycoside modulated cAMP and intracellular Ca^2+^ levels in an A_2B_AR-dependent manner, which accompanied by the suppression of p38 MAPK and ERK1/2 phosphorylation, and blocked cell cycle progression. Okh induced mitochondrial dysfunction, characterized by increased ROS production and loss of the mitochondrial membrane potential (ΔΨm), which led to the upregulation of APAF-1 and cytochrome C, activation of caspases-9 and -3, and initiation of apoptosis. The antitumor potential of Okh was confirmed in a 3D culture of MDA-MB-231 cells and was more significant than those of another A_2B_AR-targeted triterpene glycoside cucumarioside A_0_-1 and cisplatin.

## 1. Introduction

Breast cancer is a significant global health issue, projected to see a nearly 40% increase in cases and a 68% increase in deaths by 2050 [1]. Triple-negative breast cancer (TNBC) accounts for about 10–20% of all breast cancers globally and is a highly aggressive subtype [2]. Approximately 40% of TNBC patients develop metastases, often to the brain, liver, and lungs, with a median survival of 11–13 months once metastatic. This high-risk form of breast cancer lacks estrogen, progesterone, and HER2 receptors that are targets for other breast cancer treatments, requiring different therapeutic approaches [3]. Besides the absence of the common breast cancer hormone receptors, TNBC is characterized by unique molecular features, including high proliferation speed due to the increased content of proliferation markers, hyperactivation of oncogenic signaling pathways such as PI3K/AKT/mTOR [4,5] and MAPK [6], and a high quantity of tumor-infiltrating lymphocytes [7] which indicate the close interinfluence of TNBC and the immune system. The discovery of the biochemical peculiarities of TNBC triggered the elaboration of novel therapeutic strategies targeting these new molecular goals, which used to make it “elusive” for earlier treatment. This transformed TNBC from one of the most lethal subtypes into a disease where personalized therapy is beginning to change the prognosis for patients.

Promising directions in research and drug development (R&D) today are defined by several key trends: precision, personalization, speed, and complexity. The following areas of modern pharmaceuticals meet these requirements: gene therapy [8,9]; adoptive cell therapies, including T-cell receptor therapy and chimeric antigen receptor T (CAR-T) therapy [10]; and therapeutic antibodies [11]. However, these directions faced some key challenges, such as the high cost of gene and cell therapies, the unknown long-term effects of many new approaches (especially gene editing), and the absence of access to these expensive innovative therapies for people worldwide. As a result, R&D area moves to the development of highly specific individual therapies, targeting the cause of diseases. In this respect the finding of new ligands for the molecular targets inherent for TNBC and differing them from normal cells becomes especially relevant.

The adenosine receptor (AR) of A_2B_ type is one of such molecular fingerprints, it is a potential therapeutic target and a prognostic biomarker of MDA-MB-231 TNBC cells due to its high-density [12,13]. Increased A_2B_AR level is associated with aggressive forms of TNBC, including basal-like breast cancer, and it also promotes immune suppression, metastasis, and tumor growth. Other various human tumor lines are also characterized by significantly higher A_2B_AR levels than normal tissues [14,15,16,17]. A_2B_AR controls the Gs/adenylate cyclase (AC)/cAMP and Gq/phospholipase C/Ca^2+^ signaling [12,18,19] pathways that affect ERK1/2-dependent cell cycle progression. Cyclin D_1_ is also modulated via the A_2B_ adenosine receptor [20,21].

The agonists of AR–NECA, BAY 60–6583, and LUF-5835 demonstrate anti-ischemic and stimulatory effects on cell proliferation. The antagonists—PSB-1115, caffeine and others—may affect the proliferation of cells with over-expressed AR. Some A_2B_AR blockers are currently undergoing clinical trials as monotherapy or combinative therapy for cancer treatment [17,19,22]. Recently, it was found that triterpene glycosides cucumarioside A_0_-1 (Cuc A_0_-1) and djakonovioside A (Dj A) may act as biased antagonists of A_2B_AR [23], that result in the suppression of the proliferation and migration, reduction or blockage of colony formation of breast cancer cells in vitro [24] and in vivo antimetastatic action [25].

**Figure 1:**
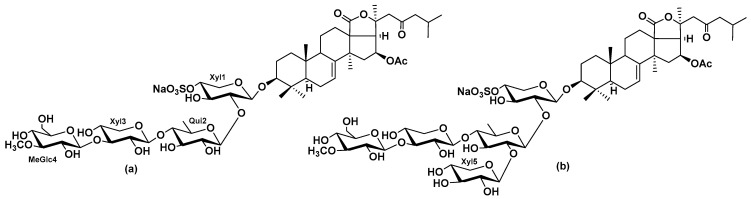
Structures of okhotoside A_1_-1 (Okh) (**a**) and cucumarioside A_0_-1 (Cuc A_0_-1) (**b**).

Another triterpene glycoside okhotoside A_1_-1 (Okh) (Figure 1a), found in *Cucumaria djakonovi* [24] and *C. conicospermium* [26] showed selective cytotoxicity against TNBC cells of the MDA-MB-231 line without significant toxicity against normal mammary MCF-10A cells. Okh structure differs from Cuc A_0_-1 (Figure 1b) by lacking the terminal xylose residue, which branches the carbohydrate chain of Cuc A_0_-1. Hence, this native glycoside represents a structural combination of the aglycone characteristic of Cuc A_0_-1 and the carbohydrate chain of Dj A. Considering that Cuc A_0_-1 and Dj A target A_2B_AR but using diverse parts of their molecules and that the cytotoxic activity of Okh was higher (IC_50_ near 2 µM), than the activities of Cuc A_0_-1 and Dj A (IC_50_ ~6 µM for each) [24] it was rather interesting to check the involvement of A_2B_AR in antiproliferative activity and anticancer effects of Okh.

Thus, the aim of this study is the investigation of A_2B_AR-dependence of Okh cytotoxic and antiproliferative action using experimental and computational approaches. Moreover, anticancer activity of Okh was examined in a 3D culture of TNBC cells of the MDA-MB-231 line.

## 2. Results

### 2.1. The Dependence of the Antiproliferative Action of Okh Against MDA-MB-231 Cell on A_2B_AR

The A_2B_AR-dependence of the antiproliferative effect of Okh on MDA-MB-231 cells was investigated in competitive long-time assays using the AR agonist NECA and the A_2B_AR antagonist PSB-1115. The effect on MDA-MB-231 cell number was studied in a 9-day test and the effect on colony formation and growth was studied in a 14-day test. The concentration of glycoside (Okh), A_2B_AR antagonist PSB-1115, and AR agonist NECA used in the tests with monolayers of MDA-MB-231 cells was 1 μM for each.

AR agonist NECA increased the number of cells and the growth of colonies by 27%. A_2B_AR antagonist PSB-1115 did not affect MDA-MB-231 cell number in agreement with the literature [27]. Nevertheless, PSB-1115 prevented the NECA-induced stimulation of cell proliferation and colony growth.

**Figure 2:**
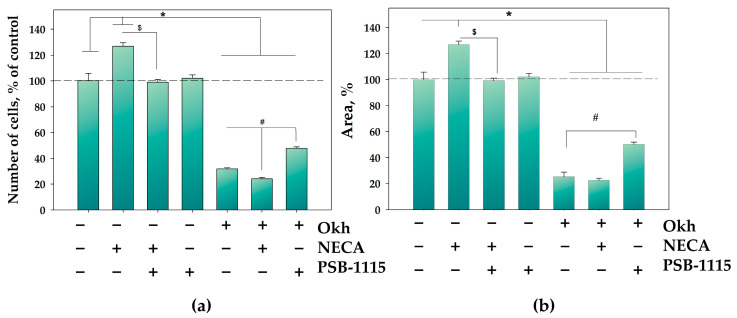
The influence of Okh (1 μM) with or without NECA (1 μM) and PSB-1115 (1 μM), on MDA-MB-231 cell proliferation for 9 days using the cell counting method (**a**). The influence of Okh (1 μM) with or without NECA (1 μM) and PSB-1115 (1 μM), on the formation and growth of MDA-MB-231 cell colonies for 14 days (**b**). * *p* < 0.05 compared to control untreated MDA-MB-231 cells; # *p* < 0.05 compared to the difference between glycosides and glycosides + NECA or glycosides + PSB-1115; $ *p* < 0.05 compared to the difference between NECA and NECA + PSB-1115.

Okh decreased the number of MDA-MB-231 cells by 68% compared to untreated cells and prevented the increase in number in NECA-treated cells. At the same time PSB-1115 reduced the antiproliferative effect of Okh (Figure 2a).

The treatment of MDA-MB-231 colonies with Okh resulted in a significant inhibition of their growth by 75% (Figure 2b). NECA did not affect the action of Okh toward colonies (Figure 2b), while PSB-1115 decreased Okh-induced inhibition of colony growth.

### 2.2. The Influence of Okh on Intracellular cAMP and Ca^2+^ Levels in MDA-MB-231 Cells

Since A_2B_AR modulation controls the Gs/adenylate cyclase (AC)/cAMP and Gq/phospholipase C/Ca^2+^ signaling [12,19] pathways, the effects of Okh on cAMP and Ca^2+^ levels in MDA-MB-231 cells were studied using an ELISA kit and a fluorescent probe FluoriCa-8 AM, respectively.

**Figure 3:**
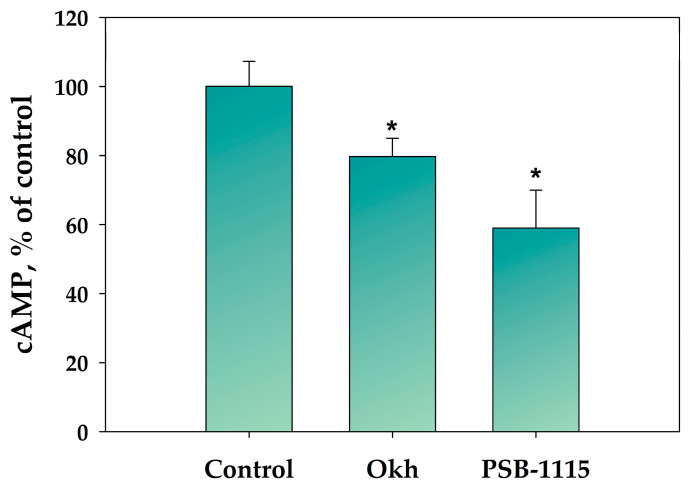
Evaluation of cAMP content in MDA-MB-231 cells after treatment with Okh (1 µM), and PSB-1115 (1 μM) for 6 h using an ELISA kit. * *p* < 0.05 compared with untreated MDA-MB-231 cells.

The treatment of MDA-MB-231 cells with Okh for 6 h decreased the cAMP level by 20% as compared to untreated cells (Figure 3). A_2B_AR antagonist PSB-1115 also decreased the cAMP level by 41%.

**Figure 4:**
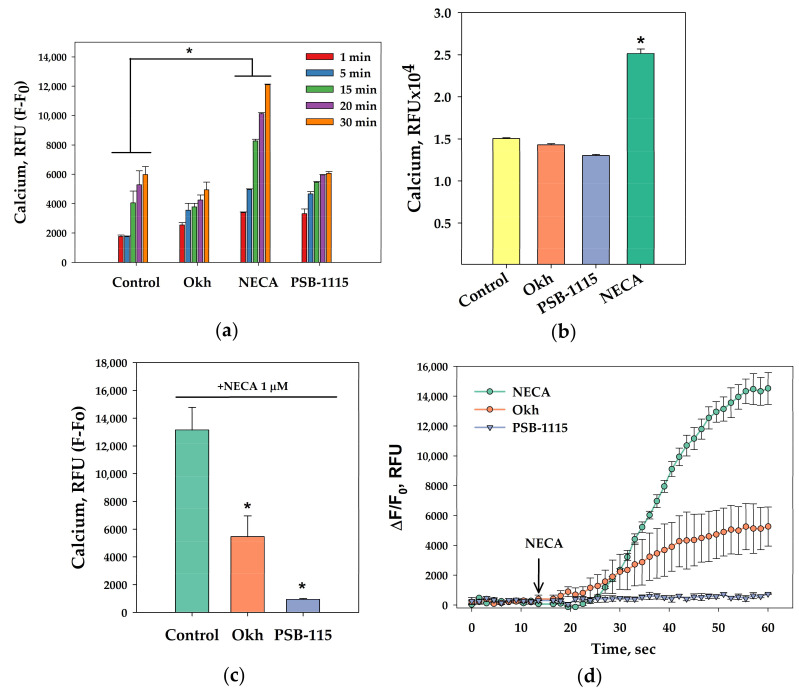
The influence of Okh on intracellular calcium levels in MDA-MB-231 cells: effect of Okh (1 µM), PSB-1115 (1 µM) and NECA (1 µM) on intracellular Ca^2+^ level in time (**a**), and at 30 min (**b**); effect of Okh (1 µM), PSB-1115 (1 µM) on Ca^2+^ level induced by NECA (1 µM) (**c**); representative curves of [Ca^2+^]i elevation induced by NECA (1 µM) alone or in the presence of Okh (1 µM) or PSB-1115 (1µM) (**d**). Data are presented as m ± SE (*n* = 6). * *p* < 0.05 compared to control (**a**,**b**) or NECA (**c**).

While Okh and PSB-1115 decreased the intracellular Ca^2+^ levels to different extent, NECA increased intracellular Ca^2+^ levels twice as compared to control (Figure 4a,b). When MDA-MB-231 cells were pre-treated with Okh or PSB-1115, the rising effect of NECA was reduced by 59% and 93%, respectively (Figure 4c,d).

Based on the attenuation of Okh’s antiproliferative effect by PSB-1115 and its inhibition of NECA-induced Ca^2+^ influx, we concluded that A_2B_AR interaction contributes to Okh’s mechanism of action. Computational modeling was subsequently applied to validate this binding.

### 2.3. In Silico Modeling of Okh Binding with A_2B_ Adenosine Receptor (A_2B_AR)

**Figure 5:**
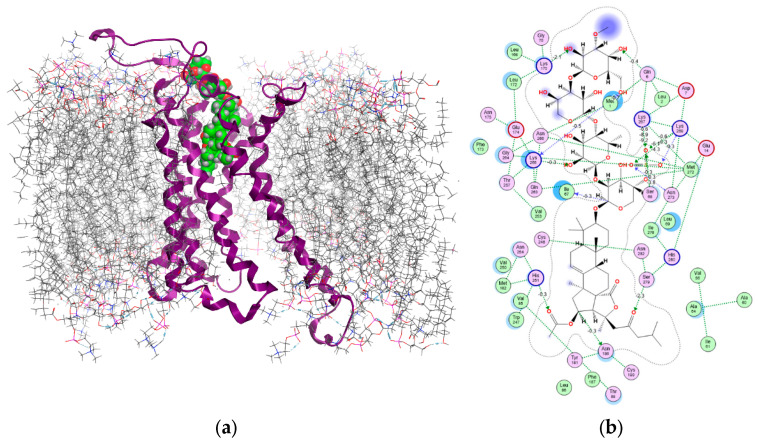
Structural diagram (**a**) of the Okh complex with A_2B_AR (binding mode A) in a lipid environment and 2D intermolecular interaction scheme (**b**). A_2B_AR is presented as a violet ribbon, asymmetric mammalian plasma membrane lipids as gray sticks and Okh as green balls. The aqueous environment and some lipid membrane components were removed for clarity.

**Figure 6:**
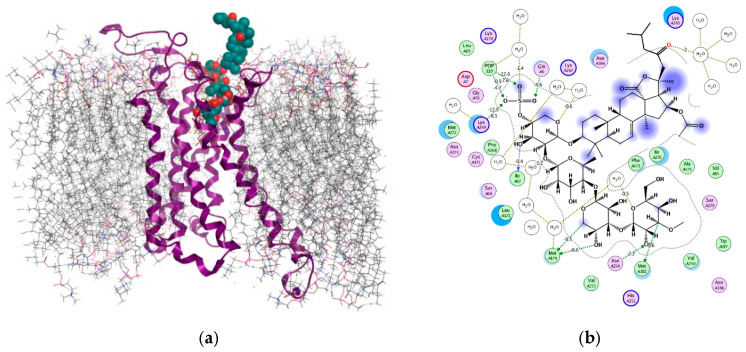
Structural diagram (**a**) of Okh complex with A_2B_AR (binding mode B) in a lipid environment and 2D intermolecular interaction scheme (**b**). A_2B_AR is presented as a violet ribbon, asymmetric mammalian plasma membrane lipids as gray sticks and Okh as dark green balls. The aqueous environment and some lipid membrane components were removed for clarity.

Previously, we employed a structure-based approach to identify the binding conformations of Cuc A_0_-1 and Dj A within the human A_2B_AR [23]. A receptor model for docking was constructed based on the cryo-EM structure of the A_2B_AR-Gs complex (PDB: 8HDP) [28]. The model was prepared by removing the Gs protein, reconstructing missing loops, and modeling disulfide bridges. Finally, it was embedded into an asymmetric mammalian membrane using CHARMM-GUI, following our established protocol [23,29,30]. In this study, we applied this approach to investigate the interaction of Okh with the A_2B_AR. Blind molecular docking revealed that the glycoside occupies the orthosteric binding site, a pocket common to receptor agonists and antagonists [31], as well as the allosteric site. Notably, the analysis identified two highly probable binding modes with comparable affinity: in mode A, the aglycone is immersed in the binding pocket, while in mode B, the carbohydrate moiety occupies the site, with docking scores of −12.83 and −10.05, respectively (Figure 5a and Figure 6a).

Binding mode A of Okh with the A_2B_AR closely resembles that of Cuc A_0_-1, with the aglycone deeply penetrating the orthosteric site [23]. However, the orientation of the carbohydrate chain differs radically. This binding mode shares several key interactions with Cuc A_0_-1, including hydrogen bonds (HBs) between the aglycone and functionally important residues [32,33]: 23-keto group with Ser279 (−2.3 kcal/mol) and 16-OAc group with His251 (−0.3 kcal/mol). His251 also mediates indirect interactions with Asn254 and Met182. Furthermore, the aglycone core forms a strong hydrophobic contact with Val250 (TM6)—a residue unique to A_2B_AR that is critical for receptor specificity—with an interaction surface of 32.13 Å^2^ and a substantial energy contribution of −4.20 kcal/mol. Concurrently, Trp247 (TM6) is engaged via tight hydrophobic interactions with the aglycone side chains, forming an interface of 32.12 Å^2^ and contributing −4.32 kcal/mol to the complex stability.

In contrast, the carbohydrate moiety of Okh forms a distinct network of hydrogen bonds between C-1 Xyl1 and Ile67 side chain, 2-OH group of Qui2 and Lys265, and 2-OH group of MeGlc4 and Lys170. The total contribution of these interactions to the complex stability was approximately −2.7 kcal/mol. Crucially, the primary stabilizing role in binding mode A is played by the glycoside’s sulfate group. This group forms an extensive network of strong hydrogen and ionic bonds with Lys267, Lys269, Asn273, and Ser68—residues that are known to be critical for high-affinity ligand binding to A_2B_AR. The individual energy contributions of these interactions were substantial: −14.79 kcal/mol, −15.68 kcal/mol, and −4.50 kcal/mol, respectively (Figure 5b).

These results indicate that this robust ionic network not only determines the specific conformation of Okh in binding mode A but is also the likely driver of its higher binding affinity and, consequently, its enhanced antagonist activity compared to other glycosides.

Given the identical carbohydrate chains of Okh and Dj A, the binding mode B of Okh shares several features with the previously reported Dj A/A_2B_AR complex [23]. In both complexes, the carbohydrate moiety occupies the receptor’s binding pocket. However, Okh’s carbohydrate chain penetrates more deeply, fully occupying the orthosteric site (Figure 6). This deeper insertion enables tighter attachment to TM6 and TM5 through HBs between 2-OH and C-3 of MeGlc4 and the highly conserved, functionally important residues Asn254 and Met182 [33,34], respectively (Figure 6b). Furthermore, the xylose residues contribute to receptor engagement, with Xyl3 and Xyl1 forming a HBs network to ECL2 and TM2 via Met179 and Ile67—residues that are also implicated in binding both nucleoside and non-nucleoside A_2B_AR agonists [35].

The analysis of non-covalent interactions reveals a critical distinction in Okh’s binding mode B compared to Dj A: the primary anchoring role is played by Lys269. This residue forms a strong HB (−12.5 kcal/mol) and salt bridges (−6.65 kcal/mol) with the Okh sulfate group, firmly securing the glycoside to ECL3. This interaction is further stabilized by Van der Waals (VdW) contacts from the Lys269 side chain (~13.8 Å^2^, −2.43 kcal/mol). Lys269, together with Lys267, is fundamental to A_2B_AR function [33] and has previously been identified as an anchoring point for selective, irreversibly bound ligands [36,37,38].

Concurrently, the sulfate group forms an additional strong HB with Gln6 in the N-terminal region (−4.6 kcal/mol). Beyond these polar interactions, the carbohydrate moiety is stabilized within the orthosteric site by an extensive network of VdW contacts with residues including Val250, Trp247, Leu272, and Ile67, with individual energy contributions ranging from −1.13 to −3.56 kcal/mol.

Furthermore, our modeling highlights the significant role of the receptor’s lipid environment. The phosphocholine head of a membrane lipid was found to fix the Okh sulfate group through a network of hydrogen and ionic bonds, contributing substantially (~−22.03 kcal/mol) to the complex stability (Figure 6b).

### 2.4. The Influence of Okh on p38/p-p38, ERK1/2/p-ERK1/2, Cyclins and CDKs Levels, and Cell Cycle Progression in MDA-MB-231 Cells

**Figure 7:**
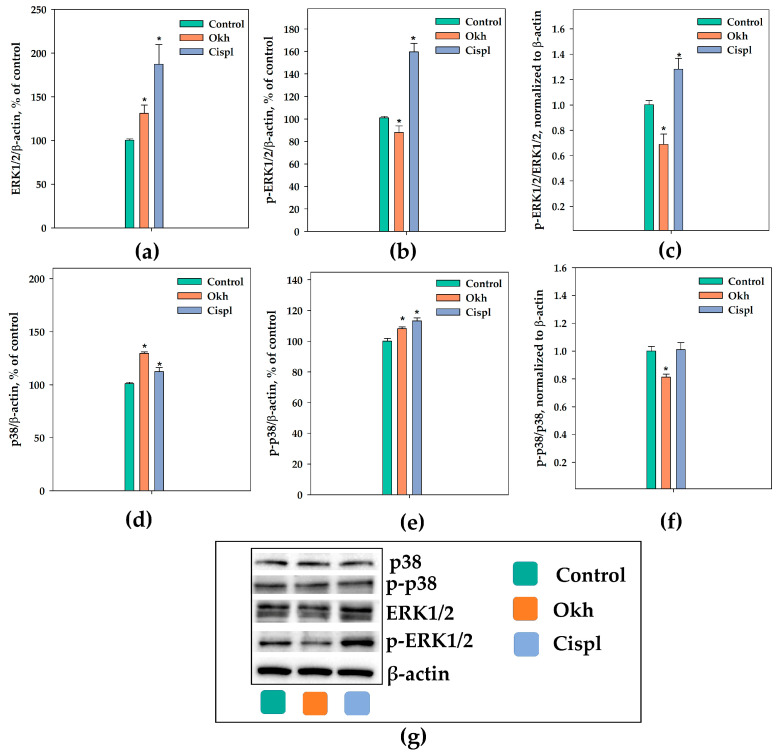
Effect of Okh (1 µM) and Cispl (10 µM) on ERK1/2/p-ERK1/2 and p38/p-p38 levels in MDA-MB-231 cells treated for 24 h. Normalization of p38 (**a**), p-p38 (**b**), and ERK1/2 (**d**), p-ERK1/2 (**e**) levels relative to the housekeeping protein β-actin. Effect of Okh (1 µM) and Cispl (10 µM) on protein level ratio p-p38/p38 (**c**) and p-ERK1/2/ERK1/2 (**f**), normalized to β-actin. Western blot results of ERK1/2, p-ERK1/2 and p38, p-p38 (**g**) levels. Protein levels were normalized to the control group (untreated cells). * *p* < 0.05 compared to control (untreated MDA-MB-231 cells).

**Figure 8:**
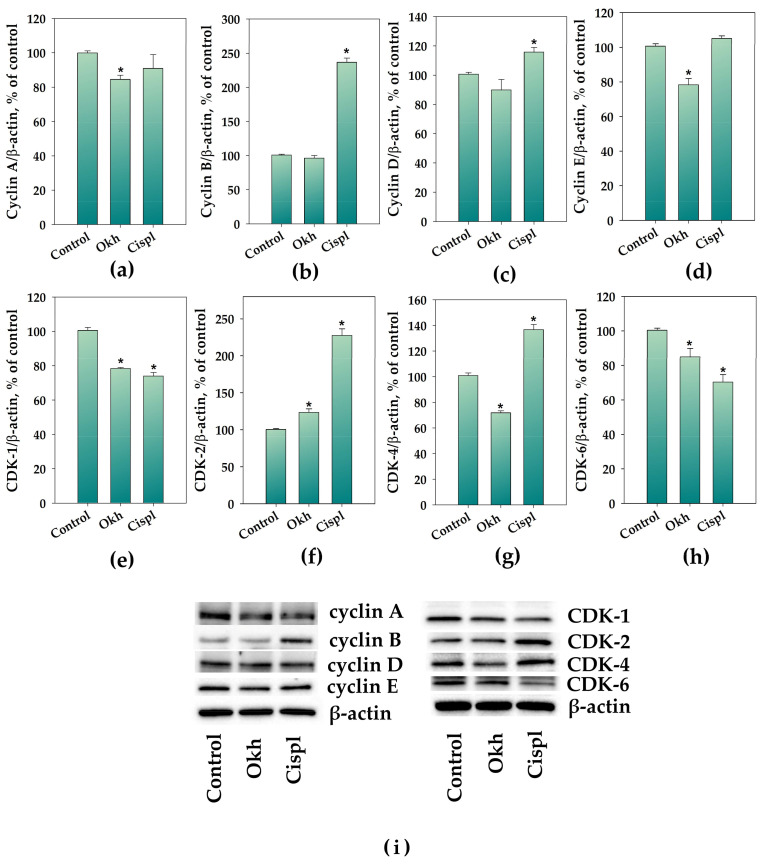
Processed data on cyclin A (**a**), cyclin B (**b**), cyclin D (**c**), cyclin E (**d**), CDK-1 (**e**), CDK-2 (**f**), CDK-4 (**g**), and CDK-6 (**h**) content in MDA-MB-231 cells treated with Okh (1 µM) and cisplatin (10 µM) for 24 h, normalized to β-actin. Visualization of cyclins A, B, D, and E content and CDK-1, -2, -4, and -6 in MDA-MB-231 cells treated with triterpene glycoside Okh A_1_-1 (1 µM) and cisplatin (10 µM) in 24 h by Western blotting (**i**). * *p* < 0.05 compared to control (untreated MDA-MB-231 cells).

The modulation of A_2B_AR activity is related to the control of cell cycle progression and the regulation of cyclin levels [16,20,39], as well as to the regulation of the MAPK pathway (p-p38, p-JNK, p-ERK) in different cell types that are characterized by a high content of this receptor [40]. The encompassing of these cascades ultimately influences cell proliferation and migration. The effect of Okh on the ERK1/2 and p38, cyclins and CDKs protein levels in MDA-MB-231 cells as well as on cell cycle progression were studied using cisplatin as a positive control (Figure 7, Figure 8 and Figures S1 and S2).

Treatment of MDA-MB-231 cells with Okh for 24 h increased the ERK1/2 protein level by 31% and decreased the level of phosphorylated ERK1/2 by 12% (Figure 7a,b). Thus, Okh decreased the p-ERK1/2/ERK1/2 ratio to 0.78 as compared to 1.0 ratio in untreated cells (Figure 7c). The same treatment increased the p38 protein level by 30% and the p-p38 level by 8% (Figure 7d,e). The p-p38/p38 protein level ratio decreased to 0.8 as compared to 1.0 in untreated cells (Figure 7f). Cisplatin (10 µM) treatment for the same time period increased the ERK1/2 protein level by 87% and p-ERK1/2 level by 60% (Figure 7a,b). The p38 protein level was increased by 12% and the p-p38 level also increased by 13% after 24 h (Figure 7d,e). Exposure of MDA-MB-231 cells to cisplatin increased the p-ERK1/2/ERK1/2 ratio to 1.28 (Figure 7c) without changing p-p38/p38 level ratio after 24 h of treatment (Figure 7f).

Okh decreased the protein levels of cyclin A by 15% and cyclin E by 20% (Figure 8a,d), while not having a statistically significant effect on cyclins B and D (Figure 8b,c) levels after 24 h of exposition to MDA-MB-231 cells. Additionally, the glycoside decreased the CDK1, CDK4, and CDK6 levels by 23%, 28%, and 15% (Figure 8e,g,h), respectively. At the same time, an increase in CDK2 level by 23% was detected (Figure 8f). A 24 h exposure of MDA-MB-231 cells to 10 µM of cisplatin resulted in an increase in cyclins B and D levels by 140% and 18% (Figure 8b,c), correspondingly, while any significant changes were not observed in the levels of cyclins A and E (Figure 8a,d). The levels of CDK2 and CDK4 were increased by cisplatin by 127% and 36% (Figure 8f,g) and the CDK1 and CDK6 levels were reduced by 26% and 30%, respectively (Figure 8e,h).

**Figure 9:**
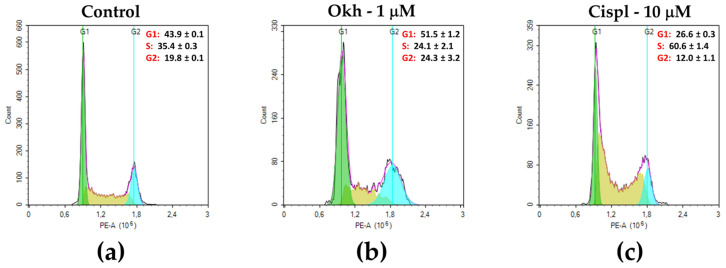
Distribution of MDA-MB-231 cells according to the phases of the cell cycle (data represented in %) in control (**a**) after treatment with Okh (1 μM) (**b**) and Cispl (10 μM) (**c**) for 24 h.

The effect of Okh on MDA-MB-231 cell cycle progression was measured after 24 h of exposition (Figure 9). The glycoside significantly changed the proportion of the G1 phase and G2/M phase cells. The percentage of G1 phase cells was increased to 51% in Okh-treated cells as compared to 44% in the untreated cells. The ratios of G2/M phase cells were 24% and 20% in Okh-treated and untreated cells, respectively. The ratio of S-phase cells was reduced under Okh action to 31.9% as compared to untreated cells 35%. Cisplatin for 24 h of exposition significantly increased the number of cells in the S-phase (Figure 9).

### 2.5. Effect of Okh on MDA-MB-231 Cell Proliferation in the 5-ethynyl-2′-deoxyuridine (EdU) Incorporation Assay

**Figure 10:**
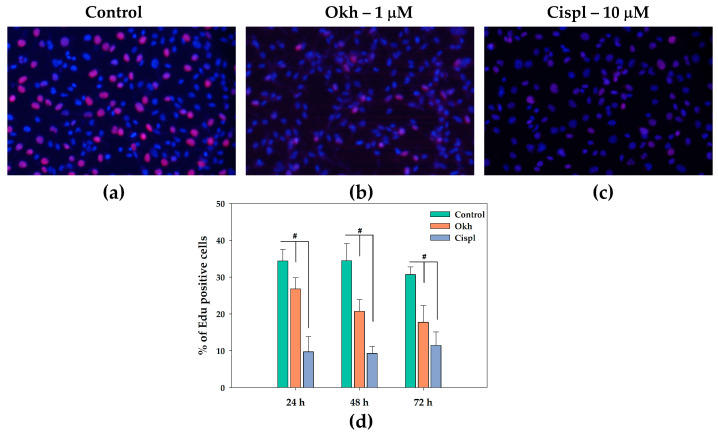
Images of EdU-treated MDA-MB-231 cell nuclei, where DNA staining is shown in blue and EdU-positive cells are shown in red. Control cells (**a**), treated with Okh (1 μM) (**b**) and Cispl (10 μM) (**c**). Data are presented as % EdU-positive cells (**d**), mean ± SEM. # *p* < 0.05 compared to control untreated MDA-MB-231 cells.

A 5-Ethynyl-2′-deoxyuridine (EdU) incorporation assay was used to verify the effect of Okh on the proliferation of MDA-MB-231 cells (Figure 10) observed in a cell counting test. Cisplatin (10 μM) was used as a positive control. The percentages of EdU-positive cells among untreated cells were 34.4% after 24 and 48 h, and 30.7% after 72 h, respectively (Figure 10), whereas in Okh-treated cells the percentages were −26.8%, 20.7%, and 17.7% after 24, 48, and 72 h, respectively. The ratios of EdU-positive cells treated with Cispl (10 μM) were 9.7%, 9.3%, and 11.5% after 24, 48, and 72 h, respectively. Thus, Okh significantly decreased the incorporation of EdU in the cells as well as arrested the cell cycle progression that confirmed its antiproliferative effect.

### 2.6. The Apoptotic Pathway in MDA-MB-231 Cells Induced by Okh

Since Gq/phospholipase C/Ca^2+^ signaling pathway triggered by A_2B_AR modulation related to ROS formation [41], which in turn causes mitochondrial membrane damage and depolarization, releasing of apoptogenic proteins and as a result triggers apoptosis [42,43], the effects of Okh on ROS levels, mitochondrial membrane potential (MMP), the content of pro-apoptotic proteins and apoptosis induction were investigated. Cisplatin was used as a positive control.

**Figure 11:**
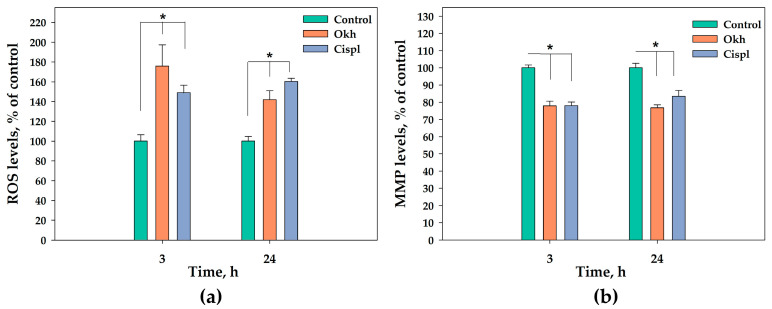
The ROS (**a**) and MMP (**b**) levels in MDA-MB-231 cells after incubation with Okh (1 µM) for 3 and 24 h measured by using fluorescent dyes: H2DCF-DA for the determination of ROS and TMRE for the determination of MMP. Cisplatin (10 μM) was used as a positive control. * *p* < 0.05, compared to the control untreated MDA-MB-231 cells.

The 3 h exposure of MDA-MB-231 cells to Okh significantly increased ROS levels by 76%, which remained elevated for 24 h. Cisplatin (10 μM) for 3 and 24 h increased ROS levels in tumor cells by 42% and 60%, respectively (Figure 11a).

The incubation of the cells with Okh for 3 and 24 h led to decreasing the MMP level by 22% and 23%, correspondingly (Figure 11b).

**Figure 12:**
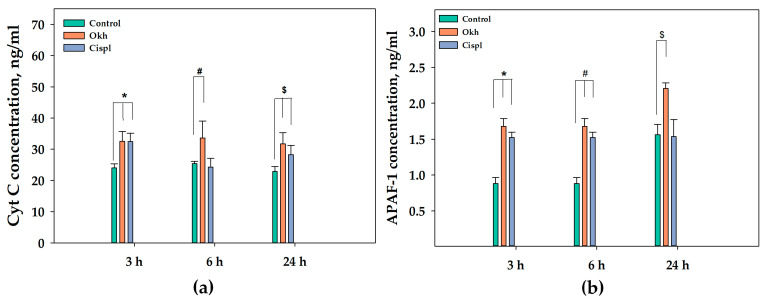
Quantitative assessment of content of cytochrome C (**a**) and APAF-1 (**b**) in MDA-MB-231 cells after the treatment with the Okh (1 µM) and cisplatin (10 µM) at the time points 3, 6 and 24 h using ELISA kits. *, #, $ *p* < 0.05, compared to the control untreated MDA-MB-231 cells.

The influence of the glycoside on cytochrom C (Cyt C) and APAF-1 levels studied with ELISA (Figure 12a,b) was the increasing of the Cyt C concentration by 35% at 3 h of exposition, and this level remained stable during 6 and 24 h (Figure 12a). Cisplatin (10 µM) increased the Cyt C concentration by 35% and 24% after 3 and 24 h of incubation, correspondingly (Figure 12a).

Okh strongly increased the APAF-1 concentrations by 90% and 99% at 3 and 24 h of exposition, respectively. While the APAF-1 level was increased only by 28% after 6 h of incubation (Figure 12b). A similar undulating pattern was observed when the MDA-MB-231 cells were treated with cisplatin (10 µM).

**Figure 13:**
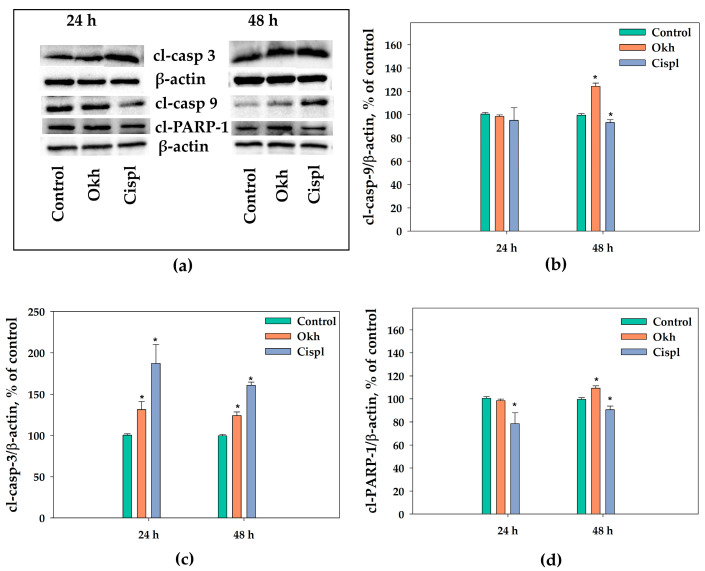
Western blot analyses (**a**) of the levels of cleaved caspase-9 (**b**), cleaved caspase-3 (**c**) and cleaved PARP-1 (**d**) in MDA-MB-231 cells treated with Okh (1 μM) and cisplatin (10 μM). β-actin was used as a protein loading control (**a**). The levels of apoptotic markers were normalized to the control group (untreated cells). * *p* < 0.05, compared to the control (untreated MDA-MB-231 cells).

The incubation of MDA-MB-231 cells with Okh during 24 and 48 h increased the level of cleaved caspase-3 by 31% and 24%, respectively (Figure 13a,c and Figure S3), and of cl-caspase-9 and cl-PARP-1 levels by 24% and 9%, respectively, after 48 h as was detected by Western blotting (Figure 13a,b,d and Figure S3). On the contrary, cisplatin (10 µM) treatment resulted in the decreasing of cl-PARP-1 content by 22% and 9% after 24 and 48 h of exposition (Figure 13d).

**Figure 14:**
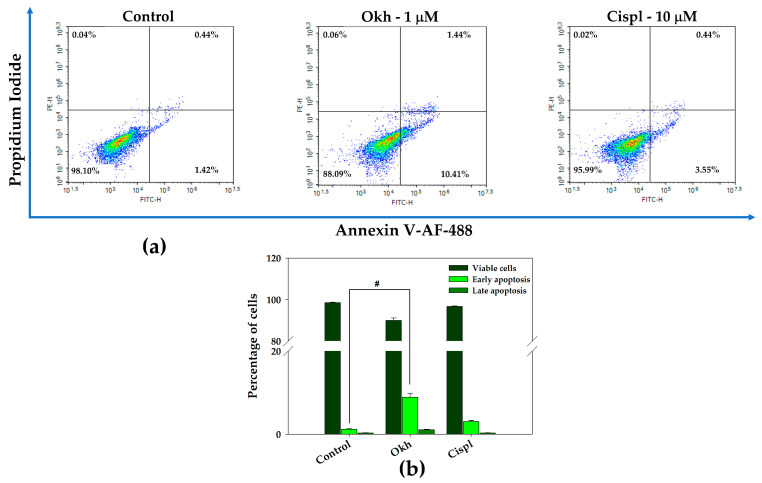
Flow cytometry analysis of apoptosis induced by Okh in MDA-MB-231 cells stained with Annexin V-AF 488/PI after 24 h of incubation (**a**). Quantitative calculation of the data obtained by flow cytometry: Control, Okh (1 μM), and Cispl (10μM) (**b**). Data are presented as means ± SEM. # *p* value < 0.05 considered significant.

The effect of Okh on the release of PS to the surface of the cytoplasmic membrane, indicating the induction of apoptosis in MDA-MB-231 cells, was assessed using the Annexin V-FITC fluorescent probe and flow cytometry technique. After 24 h of incubation the ratio of earlier apoptotic cells increased to ~10% (Figure 14).

All these data indicated that Okh demonstrated cytotoxic and antiproliferative properties in a monolayer culture of MDA-MB-231 cells and it was interesting to check its anticancer potential using 3D cultured cells.

### 2.7. The Anticancer Effect of Okh in 3D Culture of MDA-MB-231 Cells

**Figure 15:**
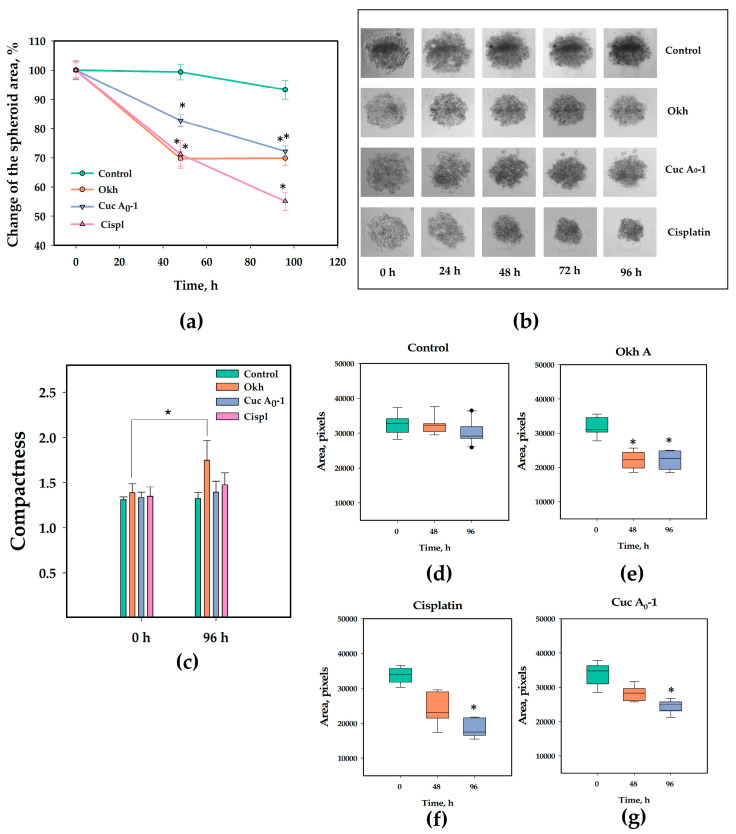
Effect of Okh (1 µM) on the MDA-MB-231 cells in 3D culture. Cuc A_0_-1 at 1 µM and cisplatin at 10 µM were used as positive controls. The measured area (**a**) and representative images of spheroids (**b**), the compactness of spheroids (**c**) during 96 h of observation. The graphs illustrate the changes in area of untreated spheroids (**d**), and spheroids treated with Okh (**e**), cisplatin (**f**), and Cuc A_0_-1 (**g**). At least 20 of the same spheroids in each variant were analyzed daily, and the data were calculated as the mean ± SEM. * indicates significance of differences (*p* < 0.05) between variants. ** indicates significance of differences (*p* < 0.01) between variants.

The anticancer action of the glycoside was investigated in relation to 3D culture of MDA-MB-231 cells which formed spheroids in agarose microwells (Figure 15). Cuc A_0_-1 was used as a positive control in addition to cisplatin, because the former in vivo anticancer effect was reported [25]. The area of spheroids and its compactness index were calculated.

The area of untreated spheroids was stable during 72 h and decreased slightly by 96 h of observations (Figure 15d). At the same time, the compactness of the untreated spheroids did not change. Treatment with Okh (1 µM) significantly reduced the area of MDA-MB-231 spheroids by 30% after first 48 h. Despite the fact that the area of spheroids remained the same after the next 48 h, the compactness index increased significantly by 26% and became more than 1.5 (Figure 15c). Compactness index is related to form factor of spheroids. A filled circle will have a compactness of 1, with irregular objects or objects with holes having a value greater than 1. Both positive controls also increased the compactness indexes of spheroids, but it was insignificant, while Okh treatment significantly increase the compactness index that indicates the increase in irregularity of spheroid form.

The treatment with Cuc A_0_-1 (1 µM) reduced spheroid area by 17% and 28% after 48 and 96 h, respectively. Treatment with cisplatin (10 µM) reduced spheroid area by 29% and 45% after 48 and 96 h, respectively.

Additionally, MTT assay was used to analyze the influence of Okh on the spheroids (Table 1).

Formazan production in Okh-treated MDA-MB-231 spheroids decreased by 14.7% in comparison with untreated spheroids. Cuc A_0_-1 did not reduced this parameter, while cisplatin also decreased it by ~16%.

It should be noted that the MTT test should be applied for spheroids and interpreted very carefully [44]. In compact spheroids, the evaluation of formazan production may be underestimated due to the difficult penetration of the MTT reagent into the spheroid with dense intercellular contacts. On the contrary, in less compact spheroids, the MTT reagent will penetrate better, that will lead to higher values of formazan production. Thus, the data about formazan production, related to viability or cell amount in untreated MDA-MB-231 spheroids may be underestimated and, respectively, the anticancer effect of Okh may be more significant, but even with this assessment its effect is stronger than that of Cuc A_0_-1.

## 3. Discussion

The experiments in monolayer and 3D cultured MDA-MB-231 cells showed the promising antitumor potential of Okh, that is provided by both antiproliferative action and apoptosis induction in breast cancer cells. Competitive functional experiments with AR agonist NECA and A_2B_AR antagonist PSB-1115 confirmed that Okh targets the A_2B_ adenosine receptor similarly to Cuc A_0_-1. However, Okh is more active than Cuc A_0_-1 due to its structural features. It possesses the same aglycone as Cuc A_0_-1 and a carbohydrate chain identical to that of Dj A [23]. Recently, it was discovered that both the glycosides are biased antagonists of A_2B_AR binding it via different modes. The aglycone of Cuc A_0_-1 drives its deep penetration to the orthosteric binding site of A_2B_AR, while Dj A binds this site through the network of intermolecular interactions formed by its sugar residues preferably. These facts forced us to conduct the in silico docking and dynamics experiments to model Okh binding with A_2B_AR. A key finding of this study is that Okh exhibits two distinct, equiprobable binding modes within the A_2B_AR orthosteric site, differentiated by a 180° rotation along its long axis. In mode A, the aglycone is inserted into the binding pocket, while in mode B, the carbohydrate chain occupies this site.

Although these modes share similarities with those of Cuc A_0_-1 and Dj A, significant differences were identified. In mode A, the linear carbohydrate chain of Okh allows its sulfate group to form an extensive, high-affinity network with key A_2B_AR residues—including Lys269, Ser68, and Asn273, in addition to the conserved interaction with Lys267 (similar to Cuc A_0_-1). This robust network, comprising multiple hydrogen and electrostatic bonds, not only alters the positioning of the carbohydrate chain at the pocket entrance compared to Cuc A_0_-1 but also explains Okh’s superior binding affinity.

In mode B, where the carbohydrate chain is fully immersed, a critical structural difference emerges. 23-keto group of the aglycone (posed extracellularly) creates an electrostatic clash with the carbonyl oxygen of Leu3, preventing it from adopting the conformation seen in the Dj A complex (which possesses a 23-OH group). Consequently, the Okh aglycone is repositioned, placing 23-keto group near a positively charged region formed by the Lys265 side chain.

The capacity of Okh to adopt dual binding modes finds support in prior structural studies of A_2B_AR. Specifically, cryo-EM structures of the partial agonist BAY60-6583 (PDB IDs 7XY6, 8HDO) demonstrate that it also binds in opposing orientations, one of which is hypothesized to be a less probable, high-energy state [28,45]. The confirmation of this phenomenon with a well-characterized ligand provides independent validation for the plausibility of our computational results with Okh.

Evaluation of the contribution of noncovalent interactions to the complex formation energies revealed that Okh binding mode A (−72.56 kcal/mol) is only marginally more favorable than mode B (−70.08 kcal/mol). This small energy difference (by approximately −2.50 kcal/mol) is consistent with the possibility of both complexes coexisting. However, a comparative analysis of the binding interfaces suggests a preference for mode A. The complex for mode A exhibits a larger ligand interface area (ΔSASA = 888.3 Å^2^ versus 630.5 Å^2^) and higher shape (S_compl_ = 0.70 vs. 0.63) and electrostatic complementarity (E_compl_ = −0.62 vs. −0.38) (Table 2). The notably lower electrostatic complementarity for mode B implies that the equilibrium likely shifts toward mode A, where the aglycone is fully immersed in the binding pocket.

Importantly, the calculated binding energies for both Okh orientations are significantly lower than those for Cuc A_0_-1 (−33.77 kcal/mol) and Dj A (−28.64 kcal/mol) [23], computationally establishing Okh as the higher-affinity ligand. This conclusion is strongly supported by our experimental data, where Okh demonstrated superior cytotoxicity, antiproliferative activity, and apoptosis induction in A_2B_AR-overexpressing MDA-MB-231 cells.

**Figure 16:**
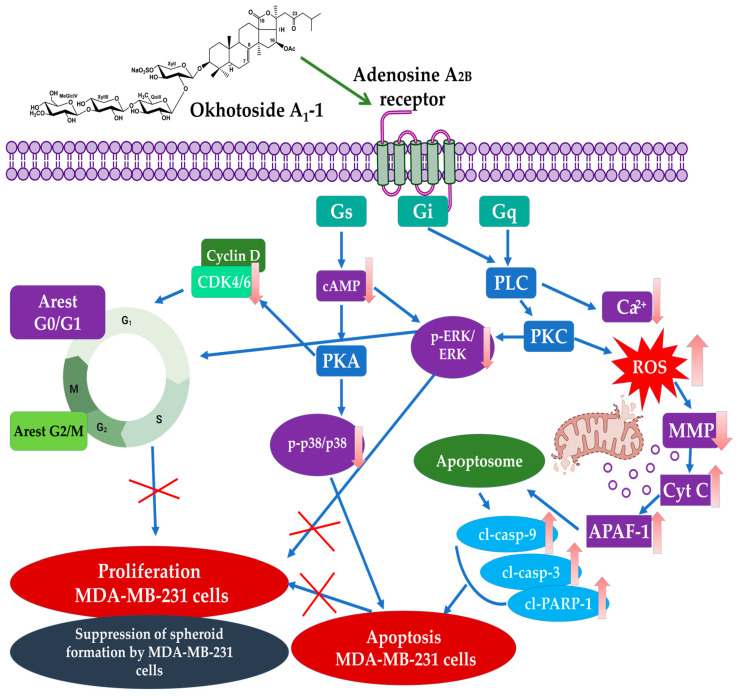
Mechanism of antitumor action of triterpene glycoside Okh in MDA-MB-231 cells via A_2B_AR correlational network.

Possible mechanisms of A_2B_AR-mediated anticancer action of Okh deduced by the analysis of obtained data is provided in Figure 16. Adenosine G protein-coupled receptors can mediate their effects through more than one signaling pathway. Activation of A_2B_AR can initiate the Gs/adenylate cyclase (AC)/cAMP and Gq/phospholipase C/Ca^2+^ signaling pathways [18]. Gq protein regulates the specific type of phospholipase C (PLC), which cleaves phosphatidylcholine (PC) to generate diacylglycerol (DAG) and phosphocholine. The resulting DAG is a secondary messenger that activates protein kinase C (PKC), which can influence ROS levels and vice versa, creating a feedback loop, affecting many cellular functions [41,46,47,48,49,50]. Increased ROS production can suppress the cell’s antioxidant defenses, causing mitochondrial damage and triggering a cascade of events that ultimately leads to a decrease in mitochondrial membrane potential [51,52,53,54]. In addition to the effect on the mitochondrial membrane, increased ROS levels can trigger the DNA damage, leading to cell cycle arrest and apoptotic cell death [55].

Our studies revealed that Okh increased ROS levels and caused mitochondrial membrane depolarization in MDA-MB-231 cells, effects that obviously occur downstream of A_2B_AR inhibition [56], because the glycoside acted similarly to the A_2B_AR inhibitor PSB-1115 and reduced the level of cAMP and blocked the calcium efflux induced by the agonist NECA in MDA-MB-231 cells [57]. ROS are one of the triggers for the initial step of mitochondrial pathway of apoptosis [58]. As a result of the launching of this cascade, cytochrome C is released from the mitochondria into the cytoplasm, where it binds to the protein APAF-1, forming a complex called the “apoptosome” followed by the activation of caspase-9, which leads to the subsequent activation of other caspases and the realization of the cell death program [59,60,61]. The increased levels of key apoptotic markers such as cl-caspase-3, cl-caspase-9, and cl-PARP-1 were detected in MDA-MB-231 cells treated with Okh, and it is consistent with previously reported data about triterpene glycosides action [25,62,63].

A_2B_AR can participate in cell cycle control via a specific mechanism involving the inhibition of MAPK signaling pathways [64] including the levels of cyclin-dependent kinase inhibitors, such as p21, which halt the cell cycle by binding to and inhibiting key cyclin-CDK complexes [65,66]. The RAF/MEK/ERK signaling pathway regulates diverse cellular processes as exemplified by cell proliferation, differentiation, motility, and survival. Notably, ERK1/2 kinases also have pro-apoptotic functions under certain conditions and enhanced ERK1/2 signaling can cause tumor cell death. Although the repertoire of the compounds which mediate ERK activation and apoptosis is expanding, and various anticancer compounds induce ERK activation while exerting their anti-proliferative effects, the mechanisms underlying ERK1/2-mediated cell death are still vague [67]. Notably, that cisplatin (in our experiments used as anticancer positive control) increased p-ERK in MDA-MB-231 cells. It was reported that activation of the ERK pathway is part of the cellular response to cisplatin treatment, which is involved in the synergistic anticancer effects of certain drug combinations, such as cisplatin and eribulin [68]. These facts evidenced the dual ambiguous role of increased ERK phosphorylation in regulation of cancer progression.

A_2B_AR signaling can influence cell cycle regulators, and targeting specific CDKs like CDK4/6, CDK2, or CDK7 is a separate but related strategy for controlling cell growth. Adenosine—the ligand of ARs—induced arrest in the cell-cycle progression in G0/G1 phase through Cdk4/cyclin D1-mediated pathway [69]. A_2B_AR antagonists induce cell cycle arrest by reducing cyclin D1/Cdk4 activity, leading to cell cycle arrest at the G1/S transition [70,71].

Our data showed that Okh influenced all these cascades and led to a decrease in the phosphorylation of ERK1/2 and p38 and significantly affected the cyclins and CDKs levels, which resulted in cell cycle arrest and proliferation blockade in the MDA-MB-231 cells. Thus, Okh can block the MDA-MB-231 cell proliferation and induce apoptotic death acting through specific membrane target, that make it a promising anti-breast cancer agent with anti-metastatic properties. High A_2B_AR level inherent for TNBC not only drives tumor proliferation and migration but also associates it with immunosuppressive tumor microenvironment (TME) and a significant downregulation of immune-related pathways [72,73]. On the other hand, inhibition of adenosine A_2A_ or A_2B_ receptors stimulates antitumor immunity and limits tumor progression [74]. Furthermore, it is known that inhibitors of A_2B_AR block the interaction of adenosine with this type of receptor, located on the surface of immune cells, that, in turn, can reduce inflammation and enhance antitumor immunity, making the immune system more effective in fighting cancer [17,19]. Thus, the low molecular weight selective A_2B_R antagonist TT-4 exerts immunosuppressive effects in the tumor microenvironment by prevention of the release of proangiogenic and immunosuppressive factors, that enhances the antitumor immune response, making this compound promising for cancer immunotherapy [75]. It is known that triterpene glycosides demonstrate immunomodulatory properties [76,77]. It was shown that cucumarioside A_2_-2, the glycoside which originated from *Cucumaria japonica*, specifically interacted with P2X receptors (predominantly P2X4) on membranes of mature macrophages that led to activation of cellular immunity [78]. Hence, a plausible hypothesis is that the anticancer efficacy of glycosides derives from their complex, simultaneous engagement of diverse membrane targets, secondary messengers, involving multiple signaling pathways. This multi-target mechanism elicits a complex organism-level response, rendering the investigation of their biological effects a highly promising research avenue. In this reason, the next step of the research should be investigation of the effects of Okh on cancer-associated immune cells in in vitro and in vivo experiments for more carefully understanding its potential as an anti-TNBC agent.

## 4. Materials and Methods

### 4.1. Reagents and Antibodies

Okhotoside A_1_-1 (Okh) were isolated from the sea cucumber *Cucumaria djakonovi* [24] and *C. conicospermium* [26] its purity was confirmed by ^1^H and ^13^C NMR spectroscopy and HR-ESI mass spectrometry. The compound was dissolved in ddH_2_O at a concentration of 1 μM and stored at +4 °C until use.

MEM medium (Biolot, St. Petersburg, Russia), fetal bovine serum (Biolot, St. Petersburg, Russia), penicilin-streptomycin (Biolot, St. Petersburg, Russia), 2′,7′-dichlorodihydrofluorescein diacetate (H_2_DCF-DA) (Sigma-Aldrich, St. Louis, MO, USA), tetramethylrhodamine ethyl ester (TMRE) (Lumiprobe, Moscow, Russia), FluoriCa-8 AM (Lumiprobe, Moscow, Russia), RIPA buffer (Sigma-Aldrich, St. Louis, MO, USA), PSB-1115 (Macklin, Shanghai, China), NECA (Calbiochem, San Diego, CA, USA), ELISA kit for cAMP (CEA003Ge, Cloud-Clone, Wuhan, Hubei, China), ELISA kit for APAF-1 (SEA054Hu, Cloud-Clone, Wuhan, Hubei, China), ELISA kit for cytochrome C (SEA594Hu, Cloud-Clone, Wuhan, Hubei, China), BSA (Sigma-Aldrich, St. Louis, MO, USA), ECL solution (Bio-Rad, Hercules, CA, USA), FluoriCa-8 AM (Lumiprobe, Moscow, Russia), Annexin-V AF 488 and propidium iodide kit (Lumiprobe, Moscow, Russia), 5-ethynyl-2′-deoxyuridine (EdU) (Lumiprobe, Moscow, Russia), Triton X-100 (Helicon, Moscow, Russia), Cu (II)-BTTAA complex (Lumiprobe, Moscow, Russia), sulfo-Cyanine5 Azide at 8 µM (Lumiprobe, Moscow, Russia), (5,6)-carboxyfluorescein succinimidyl ester (CFDA SE) dye (LumiTrace CFDA SE kit; Lumiprobe, Moscow, Russia) were used. Primary antibodies (Affinity, Shanghai, China), titer 1:1000, were used in the experiments: monoclonal mouse antibodies against p38 MAPK; polyclonal rabbit antibodies against phospho-p38 MAPK; polyclonal rabbit antibodies against caspase-9, polyclonal rabbit antibodies against caspase-3, polyclonal rabbit antibodies against PARP-1, polyclonal rabbit antibodies against ERK1/2; polyclonal rabbit antibodies against phospho-ERK1/2. Monoclonal mouse antibodies against beta-actine, titer 1:1000, secondary goat antibodies labeled with horseradish peroxidase against rabbit, titer 1:10,000 and secondary goat antibodies labeled with horseradish peroxidase against mouse, titer 1:10,000 (Cloud-Clone, Wuhan, Hubei, China) were used.

### 4.2. Cell Lines and Culture Conditions

The human triple negative breast adenocarcinoma MDA-MB-231 (HTB-26™) cell lines were sourced from the American Type Culture Collection (ATCC, Manassas, VA, USA). MDA-MB-231 cells were maintained as a monolayer under standard incubation conditions (37 °C, 5% CO_2_) in MEM medium enriched with 10% fetal bovine serum and 1% penicillin-streptomycin.

### 4.3. Analysis of ROS and MMP Levels

Reactive oxygen species (ROS) and mitochondrial membrane potential (MMP) levels were evaluated in MDA-MB-231 cells. Cells were plated at a density of 6 × 10^3^ cells per well in 96-well plates and allowed to adhere overnight (24 h). Subsequently, the cells were exposed of the glycoside for durations of 3 or 24 h. ROS production was quantified by adding 20 μL of H_2_DCF-DA solution to achieve a final concentration of 10.0 μM. To measure MMP levels, TMRE was added at a final concentration of 500 nM. Fluorescence measurements were obtained using the PHERAstar FS high-speed plate reader (BMG Labtech, Ortenberg, Germany), with excitation at 485 nm and emission at 518 nm, after 30 min incubation at 37 °C in the dark. Data processing was performed with MARS Data Analysis software (version 3.01R2), and results were presented as percentages relative to the positive control.

### 4.4. Modeling and Molecular Docking of Okh Binding with A_2B_AR in Lipid Environment

The A_2B_AR model, embedded within an asymmetric mammalian plasma membrane, served as the receptor for docking. A blind molecular docking procedure was performed using MOE 2019.01 CCG software [79] to identify the most plausible binding modes and conformations of the glycoside. The protocol involved initial protein/ligand docking with the London dG scoring function (generating 30,000 poses), followed by post-docking refinement using the Affinity dG scoring function. The Okh molecule was positioned according to the most energetically favorable pose from the docking results. The starting system was protonated at pH 7.0 using the Amber14:EHT force field within MOE 2019.01 CCG, with partial charges for the glycoside calculated using the semiempirical molecular orbital suite MOPAC 7. The system was solvated with approximately 43,600 TIP3P water molecules [80] and 0.1 M KCl (resulting in 42–64 K^+^ and 6–10 Cl^−^ ions, depending on the specific system).

All-atom molecular dynamics (MD) simulations were carried out for 20 ns using the MOE 2019.01 CCG software package [79]. The simulations were performed at constant pressure (1 atm) and temperature (300 K) with a 2 fs time step. The Amber14:EHT force field was used for the protein [81] and lipids [82], while water was modeled with TIP3P [80]. The simulation protocol included steps of energy minimization, heating, and equilibration. Finally, the contributions of non-covalent intermolecular interactions to the free energy of complex formation were estimated.

### 4.5. Enzyme-Linked Immunosorbent Assay (ELISA)

MDA-MB-231 cells were seeded in 6-well plates at a density of 5 × 10^4^ cells per well and incubated for 24 h at 37 °C in a 5% CO_2_ atmosphere until full adhesion. Subsequently, the cells were treated with 1 μM of Okh or 1 of μM PSB-1115 for 6 h. Untreated cells served as the control group. Cell lysis was performed by adding RIPA buffer, followed by centrifugation at 10,000× *g* for 15 min at 4 °C. The supernatants were collected and immediately analyzed using commercial ELISA kits for cAMP, APAF-1, or Cytochrome C, in accordance with the manufacturer’s protocols.

### 4.6. Calcium Level

MDA-MB-231 cells were seeded in 96-well plates at a density of 1 × 10^4^ cells per well in MEM medium and incubated overnight at 37 °C under a 5% CO_2_ atmosphere. Following incubation, the cells were washed once with Hank’s Balanced Salt Solution (HBSS) at pH 7.4 and loaded with 5 μM FluoriCa-8 AM ester in the same buffer. After loading, the cells were further incubated for 40 min at 37 °C in 5% CO_2_, then washed with dye-free HBSS. The cells were subsequently treated with 1 μM of Okh and 1 μM of PSB-1115 for 30 min at 37 °C under 5% CO_2_. The standard agonist NECA (at a final concentration of 1 μM) was then added to each well via a robotic microinjector to stimulate intracellular Ca^2+^ elevation, following baseline recording. Fluorescence intensity was measured using a PHERAstar FS plate reader (BMG Labtech, Ortenberg, Germany), with excitation at 490 nm and emission at 510 nm. Data analysis was performed using MARS Data Analysis software version 3.01R2 (BMG Labtech, Ortenberg, Germany).

### 4.7. Cell Cycle Analysis

The experimental procedure followed a previously described protocol. MDA-MB-231 cells were seeded at a density of 3 × 10^4^ cells/mL in a 12-well plate and allowed to attach for 24 h. Cells were then treated for 24 h with either 1 μM of Okh or 10 μM of cisplatin. Subsequently, the cells were harvested, washed twice with ice-cold phosphate-buffered saline (PBS), and fixed overnight in 70% ethanol at 4 °C. Following fixation, the cells were incubated with RNase A for 1 h at 37 °C and stained with propidium iodide solution for 5 min in the dark. Finally, samples were analyzed using a NovoCyte flow cytometer (Agilent, Santa Clara, CA, USA).

### 4.8. Western Blotting

MDA-MB-231 cells were plated at a density of 1 × 10^6^ cells per well in Petri dishes and incubated for 24 h to allow adhesion. Subsequently, the cells were treated with 1 μM Okh or 10 μM of cisplatin and further incubated for an additional 24 h or 48 h. The cells were then harvested and lysed utilizing RIPA buffer. Protein extracts were separated via electrophoresis on a Precast gel Plus Tris-Gly 4–15% gel (WSHT, Shanghai, China) and subsequently transferred onto a 0.45 μm PVDF membrane (Merk Millipore Ltd., Carrigtwohill, Irland). The membrane was blocked with 5% BSA solution for 1 h, followed by overnight incubation with primary antibodies at 4 °C and a 1-h incubation with secondary antibodies at room temperature. Protein detection was achieved using ECL solution and visualized with a ChemiDoc MP imaging system (Bio-Rad, Hercules, CA, USA).

### 4.9. Apoptosis Analysis

Apoptosis was assessed by detecting phosphatidylserine exposure on the cell surface membrane via flow cytometry employing Annexin-V-AF 488/propidium iodide (PI) dual staining. MDA-MB-231 cells were seeded at a density of 3 × 10^4^ cells/mL in 12-well plates and treated with 1 μM of the triterpene glycoside Okh or 10 μM of cisplatin for 24 h. Subsequently, cells were detached using trypsin-EDTA solution, labeled with the Annexin-V AF 488 kit and propidium iodide, and examined using a NovoCyte flow cytometer (Agilent, Santa Clara, CA, USA).

### 4.10. EdU Incorporation Assay

Cells were seeded in a 96-well plate and exposed to the compound at a concentration of 1 μM for durations of 24, 48, or 72 h. Prior to the end of the incubation period, 5-ethynyl-2′-deoxyuridine (EdU) dissolved in deionized water (dH_2_O) at 10 μM was added to each well and incubated for 2 h. Cells were then stained according to the manufacturer’s protocol. The cell monolayer was washed three times with phosphate-buffered saline (PBS) and permeabilized using 0.2% Triton X-100 in PBS for 30 min at room temperature. Subsequently, the cells underwent a click reaction staining with 10 mM ascorbic acid, 2 mM Cu(II)-BTTAA complex, and 8 μM sulfo-Cyanine5 Azide in PBS for 30 min at room temperature in the dark. Cells were washed twice with PBS, then stained with DAPI solution, and imaged under a fluorescence microscope. Images were captured at the same positions with excitation/emission wavelengths of λex = 630 nm and λem = 670 nm for sulfo-Cyanine5, and λex = 358 nm and λem = 461 nm for DAPI, using an MIB-2-FL fluorescent microscope (Lomo Microsystems, St. Petersburg, Russia). At least four positions per well were imaged, yielding approximately 15 images per incubation condition. The number of cells and EdU-positive cells per image was quantified using stardist for initial nucleus segmentation and CellProfiler for cell counting. The percentage of EdU-positive cells for each condition was subsequently determined.

### 4.11. Cell Counting Assay

MDA-MB-231 cells were seeded in 12-well plates at 100 cells per well in MEM medium and incubated overnight at 37 °C under a 5% CO_2_ atmosphere. The cells were then treated with 1 μM Okh, 1 μM PSB-1115, or 1 μM NECA. Incubation continued for 9 days in a CO_2_ incubator at 37 °C, after which cells were detached and counted using an inverted microscope (AxioVert, Carl Zeiss, Göttingen, Germany).

### 4.12. Colony Formation Assay

MDA-MB-231 cells were plated in 6-well plates at 150 cells per well and incubated overnight to allow attachment. Subsequently, the cells were treated with 1 μM Okh, 1 μM PSB-1115, and 1 μM NECA, either individually or in combination, and incubated for 14 days in a CO_2_ incubator. Colonies were fixed with methanol, stained with Giemsa reagent, and counted using a BIO-PRINT-Cx4 Edge-Fixed Pad-Container (Vilber, Collegien, France) with Bio-Vision Software user and service manual-v18.01 (Vilber, Collegien, France). Analysis of the colony-overgrown areas per well was performed using the free image processing software Fiji (ImageJ, version 1.53t, Wayne Rasband and contributors, National Institutes of Health, Bethesda, MD, USA). Results are expressed as mean values as a percentage of the control.

### 4.13. Three-Dimensional Culture of MDA-MB-231 Cells

The MSLA stamps for MDA-MB-231 spheroid formation were kindly provided by Mr. Artem Minin from M.N. Mikheev Institute of Metal Physics UB RAS (Ekaterinburg, Russia). MDA-MB-231 cell spheroids were generated in agarose microwells following a previously established procedure. In brief, 2.8% agarose heated to 60 °C was dispensed into each well of a 96-well plate, and MSLA-printed stamps featuring microcylinders were inserted and extracted once the agarose had solidified. Each well comprised 34 microwells with a 300 μm diameter. MDA-MB-231 cells were seeded at a density of 2 × 10^4^ cells per well and subjected to gentle centrifugation to facilitate cell settlement into the microwells. After 24 h, 1 μM Okh was introduced, and daily monitoring was conducted utilizing an MIB-2-FL fluorescence microscope (Lomo Microsystems, St. Petersburg, Russia). The spheroid area and compactness were quantified employing CellProfiler software version 4.2.8. A minimum of 20 equivalent spheroids per condition were evaluated daily, with results expressed as mean ± SEM. Compactness was calculated as Perimeter^2^/4*π*Area [83].

### 4.14. Statistical Analysis

All experiments were conducted in triplicate. Data underwent statistical evaluation employing one-way ANOVA tests. Results are presented as mean ± SEM, with *p* ≤ 0.05 considered indicative of statistical significance. All analyses were carried out utilizing SigmaPlot 14.0 software (Systat Software Inc., San Jose, CA, USA).

## 5. Conclusions

This study identifies the triterpene glycoside Okh as a novel and potent dual-action anticancer agent against triple-negative MDA-MB-231 breast cancer cells. We have delineated a comprehensive mechanism of action, beginning with its specific targeting of the A_2B_ adenosine receptor (A_2B_AR), a receptor overexpressed in this aggressive cancer subtype. A_2B_AR antagonism by Okh initiates a downstream signaling cascades that converge on two critical cellular processes: induction of mitochondrial apoptosis, and inhibition of proliferation and cell cycle progression. A surge in intracellular ROS caused by the glycoside, resulting in mitochondrial membrane depolarization, that launch the intrinsic apoptotic pathway. Concomitantly, Okh-mediated A_2B_AR blockade suppresses key oncogenic signaling pathways, as evidenced by reduced phosphorylation of ERK1/2 and p38 MAPK. This, coupled with a significant modulation of cyclins and CDKs, leads to cell cycle arrest and a blockade of proliferation. The specificity of this mechanism is confirmed by competitive binding assays, where Okh acts as a functional A_2B_AR antagonist. Notably, Okh exhibits superior activity compared to the related glycosides Cuc A_0_-1 and Dj A, due to “hybrid” structure, which combines the aglycone of one with the carbohydrate chain of another. Furthermore, the significant antitumor and anti-proliferative efficacy of Okh was validated not only in monolayer cultures but, more importantly, in a physiologically relevant 3D tumor spheroid model, where it effectively disrupted spheroid growth and integrity. The computational results confirm Okh’s status as a high-affinity ligand for the A_2B_AR, characterized by the lowest binding energy and the unique capability to adopt dual binding modes.

## Figures and Tables

**Table 1:** Effects of Okh (1 μM) and Cuc A_0_-1 (1 µM) on formazan production in MDA-MB-231 5-day spheroids.

Sample	Formazan Production, % of Untreated Cells
Untreated cells	100.0 ± 8.2
Okh (1 µM)	85.3 ± 6.3 *
Cuc A_0_-1 (1 µM)	96.9 ± 11.8
Cispl (10 µM)	83.9 ± 1.7 *

* *p* < 0.05 compared with untreated cells.

**Table 2:** Total SASA loss under complex formation.

Okh	ΔSASAL, Å2	ΔSASAR, Å2	S_compl_	E_compl_
binding mode A	888.3	1117.2	0.7	−0.62
binding mode B	630.5	739	0.63	−0.38

ΔSASAL—the ligand SASA loss in complex formation, ΔSASAR—the receptor SASA loss in complex formation. S_compl_—the total shape complementarity at the interface (higher values are more complementary). E_compl_—the electrostatic complementarity (negative values are more complementary).

## Data Availability

The original data are available from the corresponding author upon request.

## Supplementary Materials

The following supporting information can be downloaded at: https://www.mdpi.com/article/10.3390/md23120456/s1, Figure S1–S3: Images of original uncropped Western blots.
