# Supplementary material for: A2BAR-Mediated Antiproliferative and Anticancer Effects of Okhotoside A1-1 in Monolayer and 3D Culture of Human Breast Cancer MDA-MB-231 Cells
Source: Mar Drugs. 2025 Nov 27;23(12):456. doi: 10.3390/md23120456 (PMC12734772; doi:10.3390/md23120456)
Supplement: Supplementary file 1 [file marinedrugs-23-00456-s001.zip › marinedrugs-4000846-supplementary.pdf]

Supplementary data content page

Title: A2BAR-mediated antiproliferative and anticancer effects of okhotoside A1-1 in monolayer and 3D culture of human breast cancer MDA-MB-231 cells

Ekaterina A. Chingizova 1, †,*, Ekaterina S. Menchinskaya 1, †, Ekaterina A. Yurchenko 1, Elena. A. Zelepuga 1, Evgeny A. Pislyagin 1, Liliana E. Nesterenko 1, Sergey A. Avilov 1, Vladimir I. Kalinin 1, Dmitry L. Aminin 1,2 and Alexandra S. Silchenko 1,*

[1] G.B. Elyakov Pacific Institute of Bioorganic Chemistry, Far Eastern Branch of the Russian Academy of Sciences, Pr. 100-letya Vladivostoka 159, 690022 Vladivostok, Russia.
[2]Department of Biomedical Science and Environmental Biology, Kaohsiung Medical University, Kaohsiung 80708, Taiwan
chingizova_ea@piboc.dvo.ru (E.A.C.); ekaterinamenchinskaya@gmail.com (E.S.M.); eyurch@piboc.dvo.ru (E.A.Yu.); zel@piboc.dvo.ru (E.A.Z.); pislyagin@hotmail.com (E.A.P.); nesterenko_le@piboc.dvo.ru (L.E.N.); avilov_sa@piboc.dvo.ru (S.A.A.); kalininv@piboc.dvo.ru (V.I.K.); daminin@piboc.dvo.ru (D.L.A.); silchenko_als@piboc.dvo.ru (A.S.S.).

[†]Contributed equally to this work
*Correspondence: chingizova_ea@piboc.dvo.ru (E.A.C.); silchenko_als@piboc.dvo.ru
Tel./Fax: +7-(423)2-31-40-50

Content:

Figure S1. Images of original uncropped Western blots: ERK1/2 and p-ERK1/2; β-actin; p38 and p-p38 for 24 h.

Figure S2. Images of original uncropped Western blots: Cyclin A, Cyclin B, Cyclin E, Cyclin D, CDK-4, CDK-6, CDK-1, CDK-2 for 24 h.

Figure S3. Images of original uncropped Western blots: cl-PARP, cl-casp 9, cl-casp 3 for 24 h and 48 h.

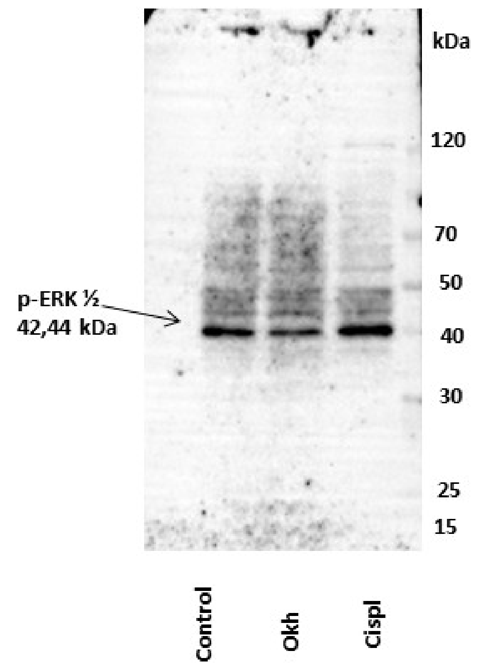

(a)

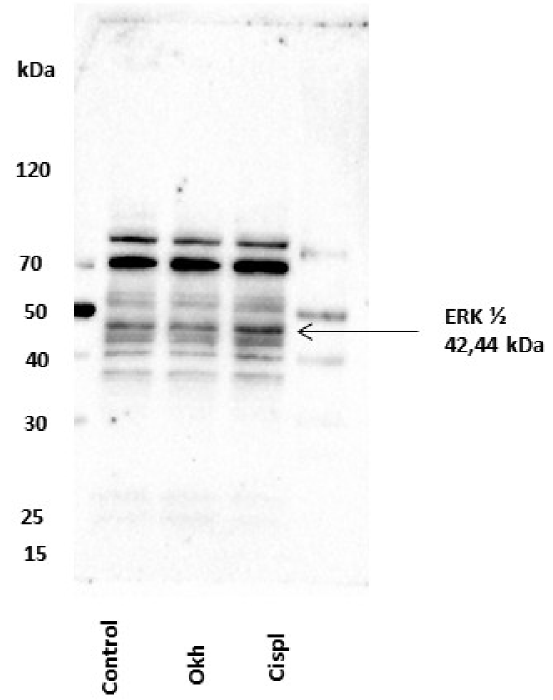

(b)

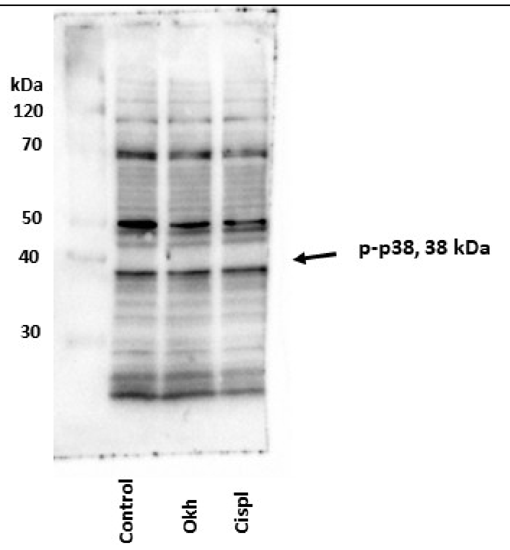

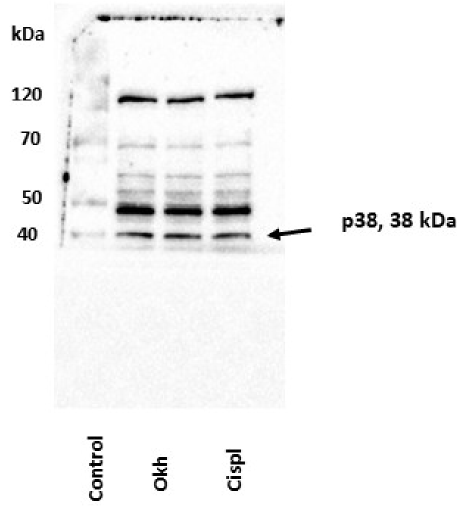

(c) **(d)**

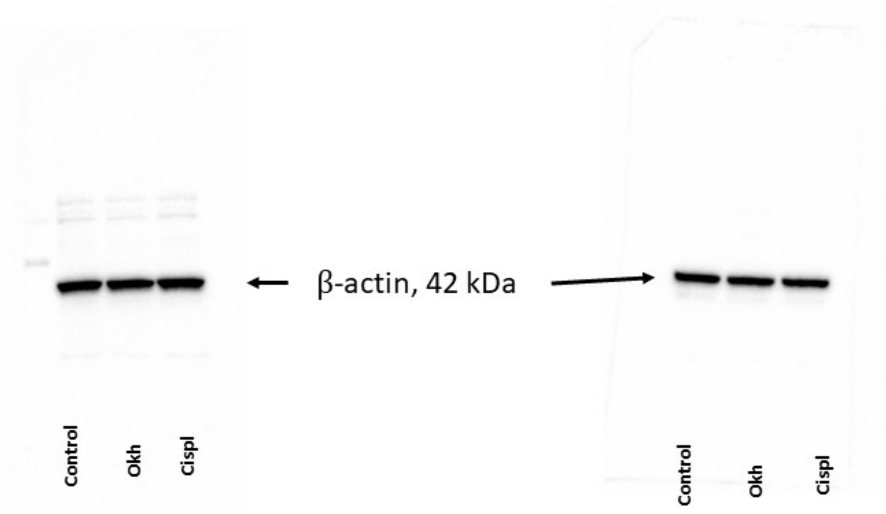

(e)

Figure S1. Images of original uncropped Western Blots displayed in this manuscript**: (a)** p-ERK1/2; **(b)** ERK1/2; **(c)** p-p38; **(d)** p38; **(e)** β-actin. Protein extracts were obtained from MDA-MB-231 cells incubated with the substances for 24 h.

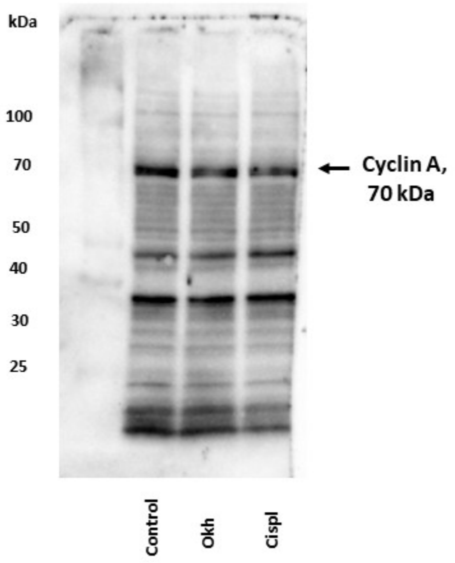
kDa
100
70
50
40
30
25
Cyclin A,
70 kDa
Control
Okh
Cispl

(a)

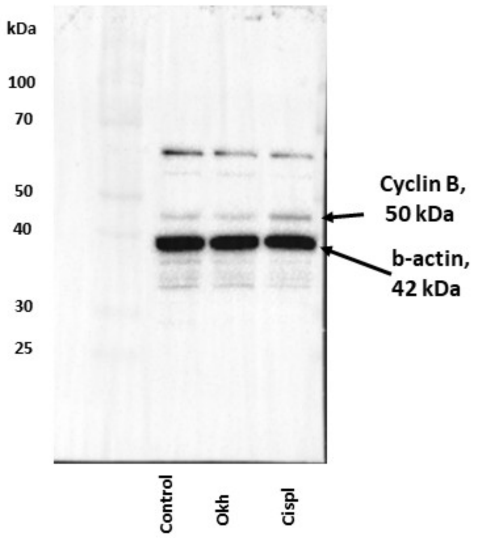
kDa
100
70
50
40
30
25
Cyclin B,
50 kDa
b-actin,
42 kDa
Control
Okh
Cispl

(b)

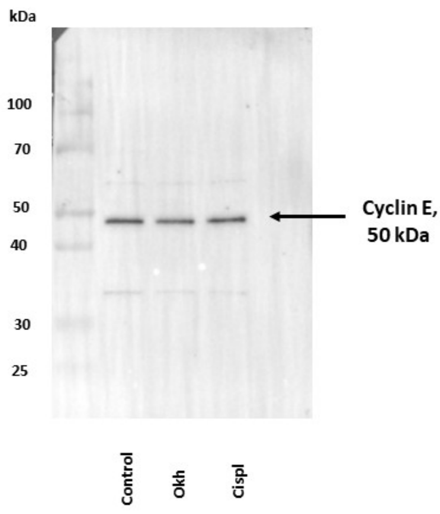
kDa
100
70
50
40
30
25
Cyclin E,
50 kDa
Control
Okh
Cispl

(c)

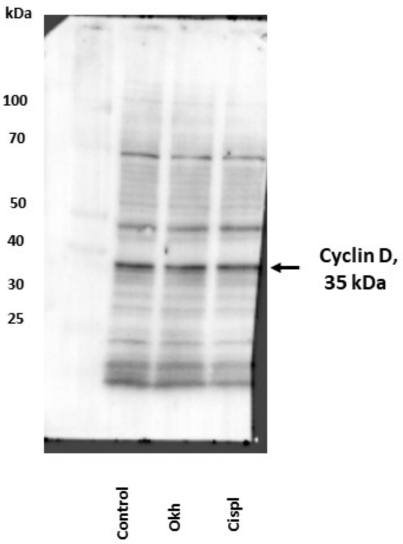
kDa
100
70
50
40
30
25
Cyclin D,
35 kDa
Control
Okh
Cispl

(d)

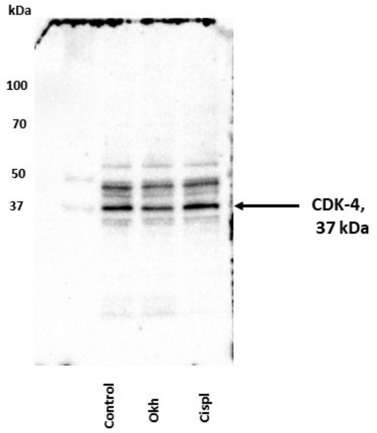
kDa
100
70
50
37
CDK-4,
37 kDa
Control
Okh
Cispl

(e)

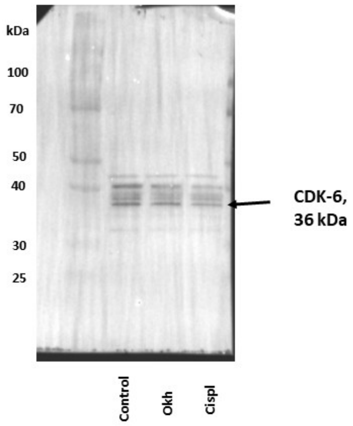
kDa
100
70
50
40
30
25
CDK-6,
36 kDa
Control
Okh
Cispl

(f)

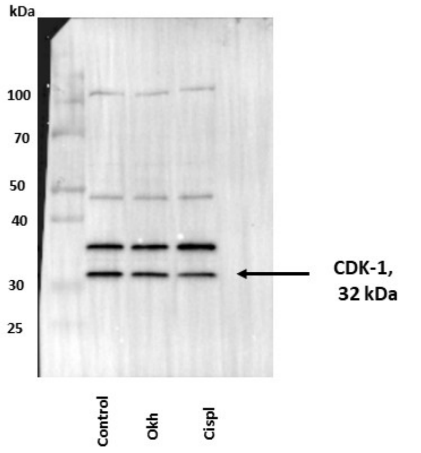

(g)

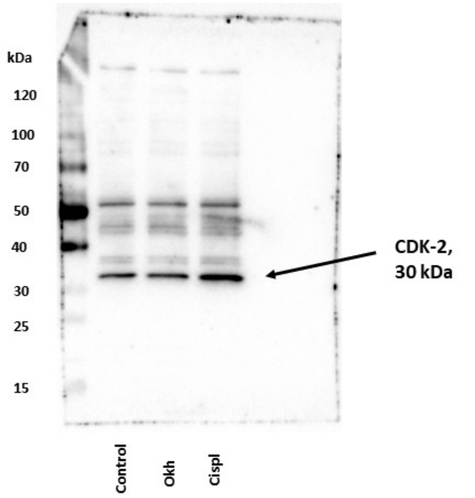

(h)

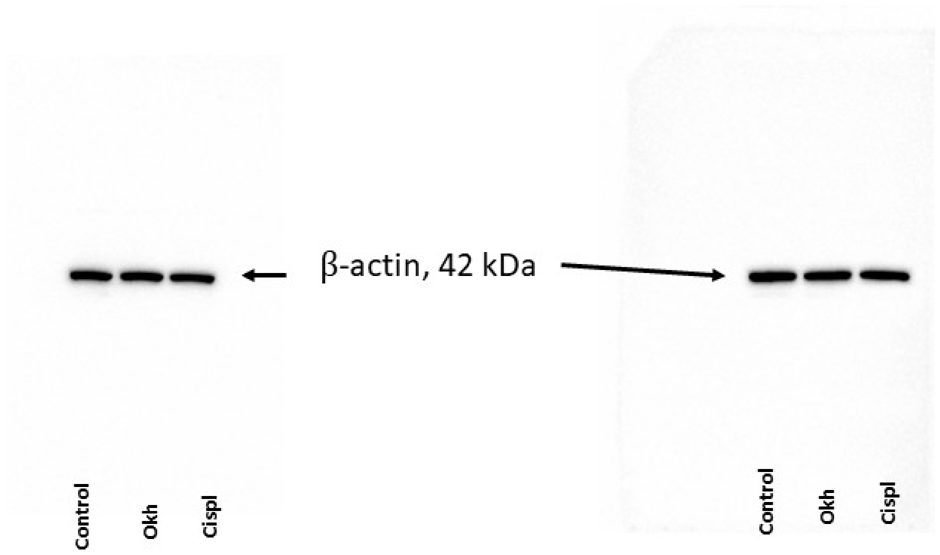

(i)

Figure S2. Images of original uncropped Western Blots displayed in this manuscript: **(a)** Cyclin A; **(b)** Cyclin B and β-actin; **(c)** Cyclin E; **(d)** Cyclin D; **(e)** CDK-4; **(f)** CDK-6; **(g)** CDK-1; **(h)** CDK-2; **(i)** β-actin. Protein extracts were obtained from MDA-MB-231 cells incubated with the substances for 24 h.

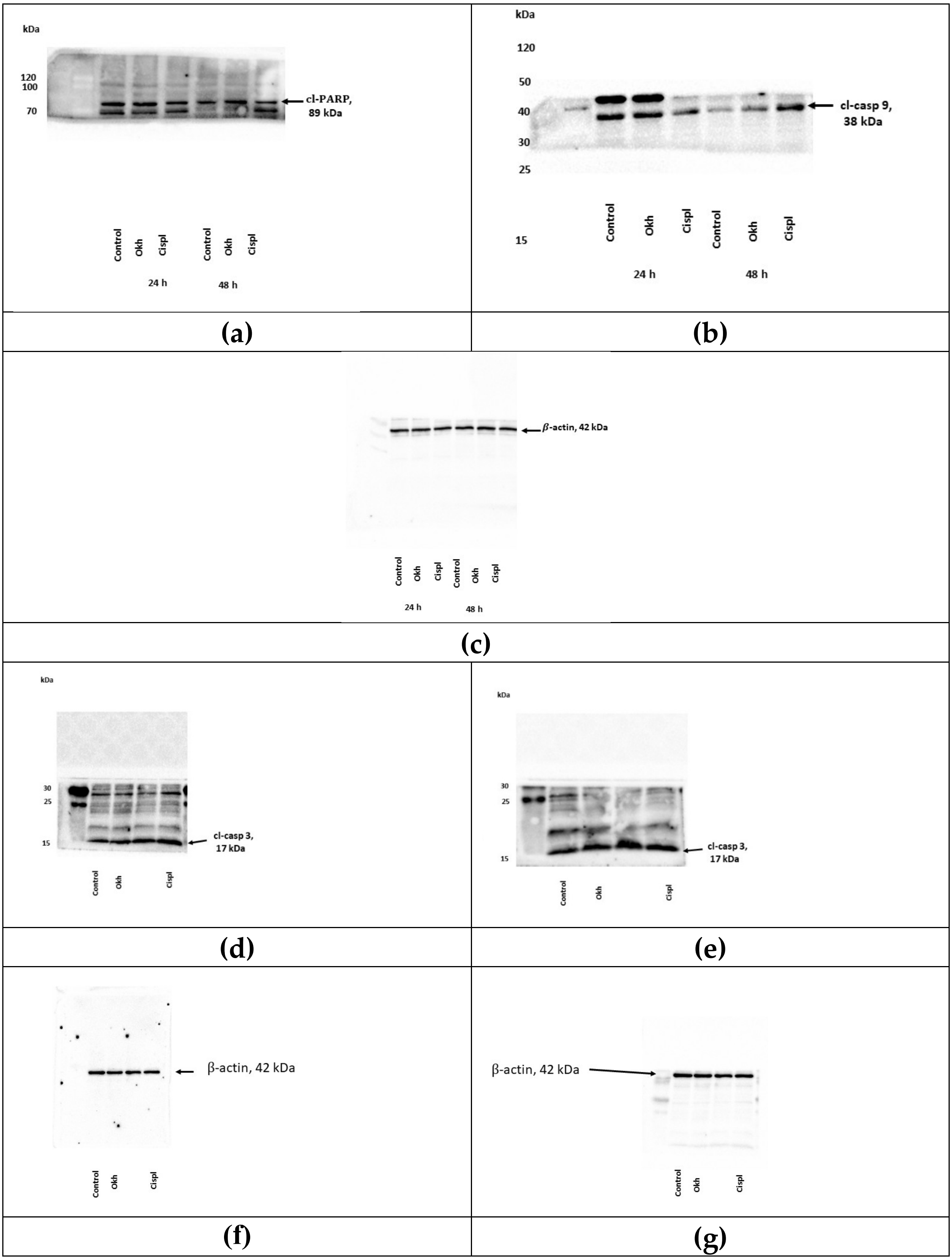

Figure S3. Images of original uncropped Western Blots displayed in this manuscript**: (a)** cl-PARP; **(b)** cl-casp 9; **(c)** β-actin; **(d)** cl-casp 3 for 24 h incubated; **(e)** cl-casp 3 for 48 h incubated; **(f)** β-actin for 24 h incubated; **(g)** β-actin for 48 h incubated. Protein extracts were obtained from MDA-MB-231 cells incubated with the substances for 24 h and 48 h.
